# MicroRNA-1 and -133 Increase Arrhythmogenesis in Heart Failure by Dissociating Phosphatase Activity from RyR2 Complex

**DOI:** 10.1371/journal.pone.0028324

**Published:** 2011-12-06

**Authors:** Andriy E. Belevych, Sarah E. Sansom, Radmila Terentyeva, Hsiang-Ting Ho, Yoshinori Nishijima, Mickey M. Martin, Hitesh K. Jindal, Jennifer A. Rochira, Yukiko Kunitomo, Maha Abdellatif, Cynthia A. Carnes, Terry S. Elton, Sandor Györke, Dmitry Terentyev

**Affiliations:** 1 The Davis Heart and Lung Research Institute, The Ohio State University, Columbus, Ohio, United States of America; 2 Division of Pharmacology, College of Pharmacy, The Ohio State University, Columbus, Ohio, United States of America; 3 Department of Medicine, Division of Cardiology, College of Medicine, The Ohio State University, Columbus, Ohio, United States of America; 4 Department of Physiology and Cell Biology, The Ohio State University, Columbus, Ohio, United States of America; 5 Department of Medicine, Cardiovascular Research Center, Rhode Island Hospital and the Warren Alpert Medical School of Brown University, Providence, Rhode Island, United States of America; 6 Department of Cell Biology & Molecular Medicine, School of Medicine of New Jersey, University of Medicine and Dentistry of New Jersey, Newark, New Jersey, United States of America; Brigham & Women's Hospital - Harvard Medical School, United States of America

## Abstract

In heart failure (HF), arrhythmogenic spontaneous sarcoplasmic reticulum (SR) Ca^2+^ release and afterdepolarizations in cardiac myocytes have been linked to abnormally high activity of ryanodine receptors (RyR2s) associated with enhanced phosphorylation of the channel. However, the specific molecular mechanisms underlying RyR2 hyperphosphorylation in HF remain poorly understood. The objective of the current study was to test the hypothesis that the enhanced expression of muscle-specific microRNAs (miRNAs) underlies the HF-related alterations in RyR2 phosphorylation in ventricular myocytes by targeting phosphatase activity localized to the RyR2. We studied hearts isolated from canines with chronic HF exhibiting increased left ventricular (LV) dimensions and decreased LV contractility. qRT-PCR revealed that the levels of miR-1 and miR-133, the most abundant muscle-specific miRNAs, were significantly increased in HF myocytes compared with controls (2- and 1.6-fold, respectively). Western blot analyses demonstrated that expression levels of the protein phosphatase 2A (PP2A) catalytic and regulatory subunits, which are putative targets of miR-133 and miR-1, were decreased in HF cells. PP2A catalytic subunit mRNAs were validated as targets of miR-133 by using luciferase reporter assays. Pharmacological inhibition of phosphatase activity increased the frequency of diastolic Ca^2+^ waves and afterdepolarizations in control myocytes. The decreased PP2A activity observed in HF was accompanied by enhanced Ca^2+^/calmodulin-dependent protein kinase (CaMKII)-mediated phosphorylation of RyR2 at sites Ser-2814 and Ser-2030 and increased frequency of diastolic Ca^2+^ waves and afterdepolarizations in HF myocytes compared with controls. In HF myocytes, CaMKII inhibitory peptide normalized the frequency of pro-arrhythmic spontaneous diastolic Ca^2+^ waves. These findings suggest that altered levels of major muscle-specific miRNAs contribute to abnormal RyR2 function in HF by depressing phosphatase activity localized to the channel, which in turn, leads to the excessive phosphorylation of RyR2s, abnormal Ca^2+^ cycling, and increased propensity to arrhythmogenesis.

## Introduction

Heart failure (HF) remains a leading cause of mortality and approximately 50% of HF patients die suddenly as a result of ventricular tachyarrhythmias stemming from the increased propensity of ventricular myocytes to generate delayed and/or early afterdepolarizations [1], [2]. The generation of arrhythmogenic afterdepolarizations in HF has been linked to extrasystolic spontaneous Ca^2+^ release from the sarcoplasmic reticulum (SR) [3], [4], [5], [6], [7]. It is believed that the increased occurrence of spontaneous Ca^2+^ release in ventricular myocytes corresponds to the abnormally high activity of ryanodine receptors (RyR2s), the SR Ca^2+^ release channels [8], [9]. Studies investigating the RyR2 properties in HF have consistently reported enhanced phosphorylation of the RyR2 either at the cAMP-dependent protein kinase A (PKA) site S-2808 [10] and/or at the Ca^2+^/calmodulin-dependent protein kinase (CaMKII) site Ser-2814 [11]. However, the molecular events leading to RyR2 hyperphosphorylation in HF remain uncertain [12], [13].

In general, enhanced phosphorylation can be explained by the increased activity of kinases and/or the decreased activity of phosphatases in the vicinity of the channel. Two phosphatases have been shown to scaffold to the RyR2 complex, PP1 and PP2A. While PP1 has been identified as the phosphatase controlling RyR2 phosphorylation at the PKA site Ser-2808 [14], PP2A appears to regulate the phosphorylation state of RyR2 at the CaMKII site Ser-2814 and at a second PKA site Ser-2030 [14], [15]. In HF, decreases in phosphatases PP1 and PP2A scaffolded to RyR2 has been suggested as an underlying cause for hyperphosphorylation of RyR2 at the PKA Ser-2808 [10] or the CaMKII Ser-2814 sites [11].

We have previously reported that phosphatase activity in the vicinity of the RyR2 channel can be regulated by the muscle-specific microRNA (miRNA) 1 (miR-1) [16]. miRNAs are a newly described class of regulators of cellular function that inhibit gene expression by targeting messenger RNAs (mRNAs) for translational repression or cleavage [17], [18]. We demonstrated that adenovirally-mediated overexpression of miR-1 promotes arrhythmogenic spontaneous Ca^2+^ release and increases CaMKII-dependent phosphorylation of RyR2 by targeting the regulatory subunit of PP2A B56α [16]. Another regulatory subunit of PP2A known to tether the PP2A catalytic subunit to the RyR2 complex, B56δ was recently validated as a target for miR-133, the second most abundant miRNA in the heart [19]. Importantly, many studies [20], [21], [22], [23], [24], [25], [26], [27] have demonstrated that the expression patterns of miRNAs are substantially altered in a number of cardiac diseases, including HF. In the present study, we utilized a canine model of nonischemic HF to test the hypothesis that the HF-related changes in RyR2 phosphorylation levels were mediated by the disruption of phosphatase activity localized to RyR2s due to enhanced expression of the two most abundant muscle-specific miRNAs, miR-1 and miR-133 [28].

## Results

### Pharmacological inhibition of phosphatase activity in control myocytes promotes diastolic spontaneous Ca^2+^ waves and delayed afterdepolarizations in the presence of the β-adrenergic agonist isoproterenol

**Figure 1:**
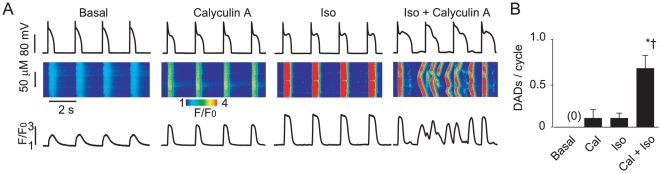
Inhibition of phosphatase activity is sufficient to promote arrhythmogenic spontaneous Ca^2+^ waves in normal paced myocytes during beta-adrenergic stimulation. A. Representative confocal Ca^2+^ images, with corresponding time dependent profiles (bottom) and membrane potential recordings (top) in control myocytes at basal conditions, preincubated with 100 nM Calyculin A for 30 min, incubated with 100 nM isoproterenol for 3 min (Iso) and challenged with Iso after 30 min incubation with Calyculin A. B. Pooled data for a number of delayed after-depolarizations due to spontaneous Ca^2+^ waves per cycle. Basal SCW frequency was 0 out of 9 cells. Statistically significant at p<0.05, vs. * Calyculin A and † vs Iso, n = 5.

First, we examined whether or not inhibition of phosphatase activity would result in the enhanced propensity of control ventricular myocytes toward generation of Ca^2+^-dependent afterdepolarizations. To examine the effects of reduced phosphatase activity on Ca^2+^ cycling and membrane potential, the PP1 and PP2A inhibitor Calyculin A [29] (100 nM, 30 min pre-incubation) was used. Control cells stimulated only with the β-adrenergic agonist, isoproterenol (Iso), displayed a moderate frequency of diastolic spontaneous Ca^2+^ waves (SCW) and delayed afterdepolarizations (DADs) (Fig. 1). However, myocytes pretreated with Calyculin A and subsequently stimulated with Iso resulted in a significant increase (∼six fold) in the rate of diastolic Ca^2+^ waves and DADs. The frequency of SCWs and DADs in myocytes treated with Calyculin A alone did not significantly differ from that observed with Iso alone. Taken together these results suggest that phosphatase activity plays a critical role in controlling the stability of intracellular Ca^2+^ cycling.

### HF myocytes display increased levels of muscle-specific miR-1 and miR-133 and decreased levels of their respective targets, regulatory (B56α and B56δ and catalytic subunits of PP2A

**Figure 2:**
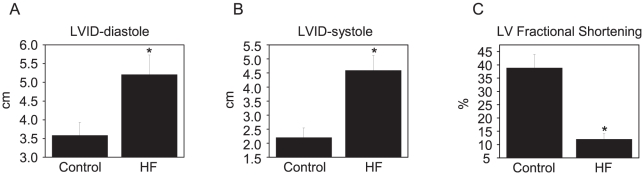
Heart failure animals have increased LV chamber size and reduced fractional shortening. A. LV internal diameter- end diastole is increased in the 4 month HF dogs. B. LV internal diameter- end systole is increased in the 4 month HF dogs. C. LV fractional shortening is reduced in the 4 month HF dogs. *p<0.05, n = 5–6.

**Figure 3:**
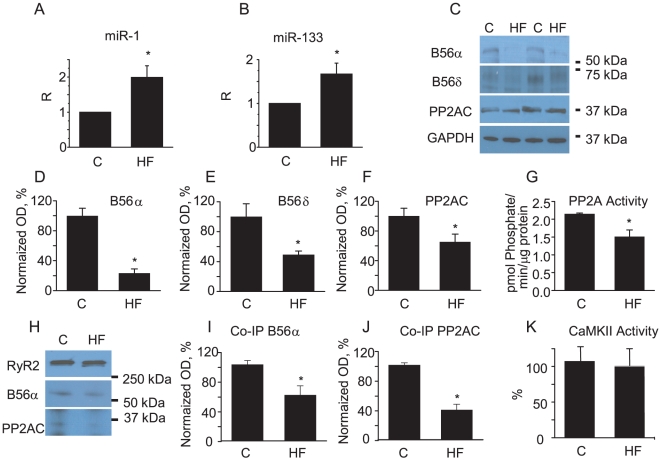
Changes in Expression Levels of miR-1 and miR-133 and their targets in canine HF Myocytes. A, B. Normalized levels of miR-1 and -133 assessed with qRT-PCR. *p<0.05, n = 5. C. Representative Western blots of putative targets of miR-1 and -133, regulatory subunits B56α, B56δ and catalytic subunit of PP2A respectively. D,E,F. Normalized optical density (OD) for B56α, B65δ and catalytic subunit of PP2A respectively; *p<0.05, n = 8. Note that anti-PP2AC antibody recognizes both α and β isoforms. G. Total PP2A activity is samples prepared from LV tissues of normal and failing hearts, *p<0.05, n = 4. H,I,J. Decrease in levels of PP2A scaffolded to RyR2 tested using coimmunoprecipitation with anti-RyR2 antibodies; (H) representative Western blots and pooled data for B56α (I) and PP2AC (J), *significantly different vs. control, p<0.05, n = 6. K. Local CaMKII activity measured in samples with immunoprecipitated RyR2s (n = 4). Local CaMKII activity levels and levels of PP2AC and B56α were normalized to the levels of RyR2s.

**Figure 4:**
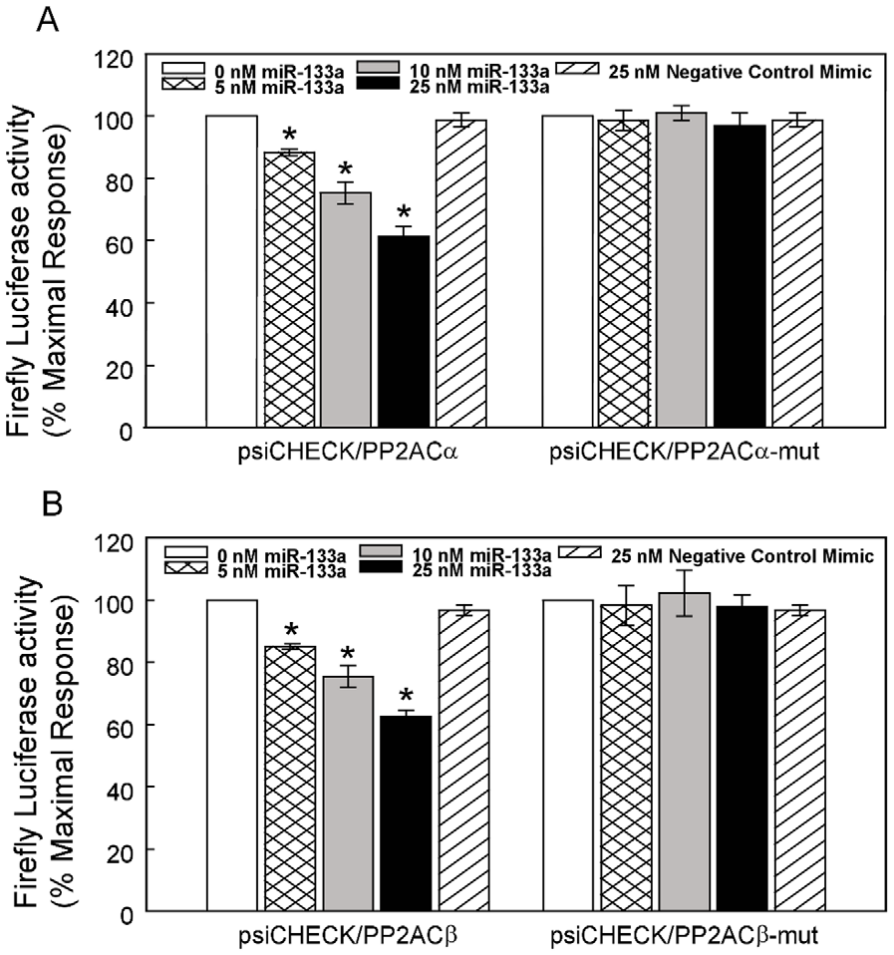
MiR-133 targets both catalytic subunits of the PP2A phosphatase. A. CHO cells were transfected with either the psiCHECK-PP2ACα or psiCHECK-PP2ACα-mut (i.e. the putative miR-133 binding has been mutated) luciferase reporter construct and miR-133, or negative control miRNA mimics at the concentrations indicated. B. Alternatively, CHO cells were transfected with either the psiCHECK-PP2ACβ or psiCHECK-PP2ACβ-mut luciferase reporter construct and miR-133, or negative control miRNA mimics at the concentrations indicated. Twenty-four hours following transfection, luciferase activities were measured. *Renilla* luciferase activity was normalized to firefly luciferase activity and mean activities ± S.E. from three-five independent experiments are shown (*p<0.05 vs. 0 nM).

**Figure 5:**
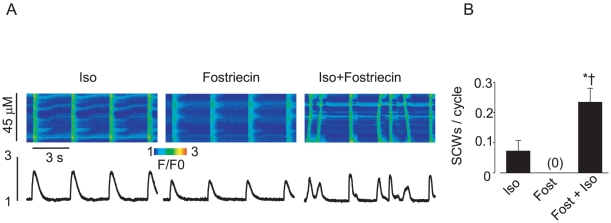
Inhibition of PP2A promotes spontaneous Ca^2+^ release under condition of β-adrenergic stimulation. A, Representative line-scan images and temporal profiles of Rhod-2 fluorescence recorded in control myocyte treated with 100 nM Iso alone, 300 nM fostriecin (30 min preincubation), a specific PP2A inhibitor, alone, and 300 nM fostriecin plus 100 nM Iso, respectively. Cells were field-stimulated at 0.3 Hz. B, Frequency of diastolic SCWs was calculated for myocytes treated with Iso alone (n = 12), fostriecin (Fost) alone (n = 11), and Fost plus Iso (n = 11). *, p<0.05 Fost plus Iso vs. Iso alone; †, p<0.05 Fost plus Iso vs. Fost alone.

HF was characterized by increased LV chamber dimensions and reduced LV fractional shortening (Fig. 2). Analysis using quantitative RT-PCR revealed that the levels of mature miR-1 and -133 were significantly increased in myocytes isolated from failing hearts when compared with controls (Fig. 3A and 3B). Western blot analysis of B56α and B56δ regulatory and catalytic subunits of PP2A, which are putative targets of miR-1 and -133 (http://www.targetscan.org), showed significant decreases in expression levels in HF vs. control myocytes (Fig. 3C,D,E,F). Consistent with the observed decrease in protein levels, total PP2A activity was also significantly reduced (30%) in HF when compared with controls (Fig. 3G). Additionally, immunoprecipitation studies demonstrated that the levels of B56α and PP2A catalytic subunits scaffolded to RyR2s were significantly decreased (i.e. ∼40% and 60% of control values respectively, Fig. 3H,I,J). Notably, CaMKII activity localized to RyR2s was not changed in HF (Fig. 3K). Targeting of the PP2A catalytic α and β subunits by miR-133 was validated using a 3′-untranslated region (3′-UTR) luciferase reporter assay (Fig. 4A,B). To test whether or not PP2A inhibition was sufficient to promote spontaneous Ca^2+^ release, myocytes were treated with a specific PP2A inhibitor, fostriecin [30]. Preincubation of myocytes with fostriecin and subsequent stimulation with Iso significantly enhanced the propensity of field-stimulated myocytes to generate pro-arrhythmic spontaneous Ca^2+^ waves. (Fig. 5A,B).

**Figure 6:**
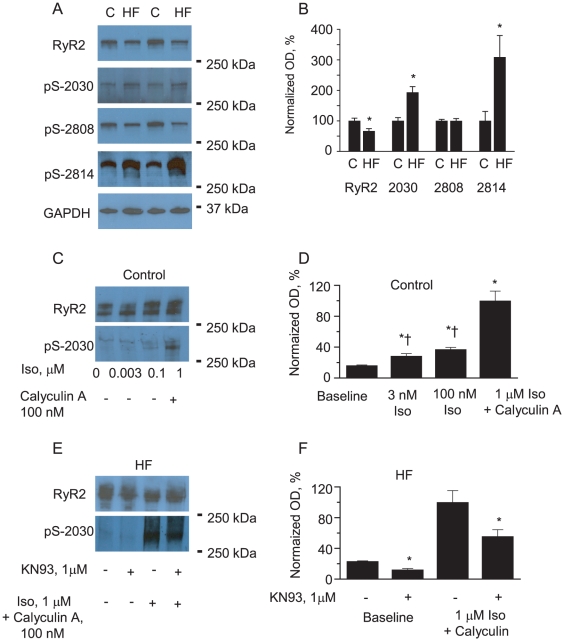
Enhanced phosphorylation of RyR2 at sites S-2814 and S-2030 in HF. A, B. Representative Western blots (A) and pooled data (B) for normalized RyR2 phosphorylation at sites - S-2030 and S-2808 and S-2814 to total RyR2 content measured in gels run in parallel. Levels of RyR2s in HF and control cells were compared using GAPDH as loading control. *p<0.05, n = 8. C.D, Representative Western blots (C) and pooled data (D) for β-adrenergic agonist Isoproterenol dose-dependence of S-2030. Signals obtained with anti pS-2030 ab were normalized to the levels of RyR2s assessed in gels run in parallel and normalized to maximum level of phosphorylation achieved by 30 min incubation of myocytes isolated from normal hearts with PP1 and PP2A inhibitor Calyculin A (100 nM) and exposed to 1 µM Iso for 3 min. *†,p<0.05 vs baseline and Iso+Calyculin respectively, n = 6. E,F. Representative Western blots and pooled data illustrating sensitivity of S-2030 to phosphorylation by CaMKII in myocytes isolated from failing hearts. Incubation with CaMKII inhibitor KN93 (1 µM, 15 min) significantly reduced S-2030 phosphorylation at basal conditions and after myocytes treatment with Iso and Calyculin A. *p<0.05 vs no KN93, n = 4.

Using phosphospecific antibodies we assessed the phosphorylation state of RyR2s in HF vs. control myocytes at three different sites, and found no changes in the RyR2 phosphorylation state at the PKA site Ser-2808. However, a 3.5-fold increase in phosphorylation was observed at the CaMKII site Ser-2814 and 2-fold increase in phosphorylation at Ser-2030 in HF myocytes (Fig. 6A and 6B). Consistent with previous reports [14], site Ser-2030 phosphorylation by PKA in response to Iso stimulation was partial (Fig. 6C,D). Therefore, to achieve maximal site Ser-2030 phosphorylation, treatment with Iso must be complemented by inhibition of phosphatase activity (Fig. 6C,D). Importantly, treatment of HF myocytes with the CaMKII inhibitor KN93 (1 µM, 15 min) significantly reduced site Ser-2030 phosphorylation levels at basal conditions, and in the presence of phosphatase inhibitor and Iso (Fig. 6E,F). Collectively, these results indicate the involvement of muscle-specific miRNAs in the regulation of CaMKII-dependent phosphorylation of the RyR2 via PP2A.

### Coordinated expression of miR-1 and miR-133 in cardiomyocytes

**Figure 7:**
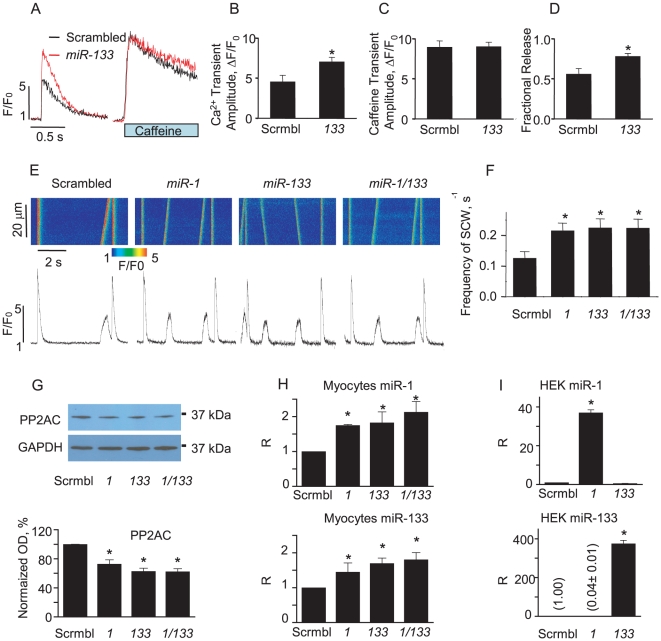
Overexpression of miR-1 and miR-133 increases the frequency of pro-arrhythmic spontaneous Ca^2+^ waves (SCW) in rat myocytes. A, Time dependent profiles of electrically- (field stimulation at 0.5 Hz) and 10 mM caffeine-evoked Ca^2+^ transients in myocytes infected with scrambled and miR-133 carrying Advs (MOI 100) after 48 hrs in primary culture. B,C,D. Pooled data for electrically and caffeine evoked Ca^2+^ transient amplitude and fractional release in miR-133 overexpressing myocytes vs. controls. E. Representative confocal Ca^2+^ images with corresponding time dependent profiles recorded in field-stimulated (0.2 Hz) myocytes in the presence of 100 nM isoproterenol infected with miR-1 and/or miR-133 Adv. F. Pooled data for the number of SCWs per second. *Statistically significant at p<0.05 vs. scrambled, n = 15–25. G. Representative Western Blots (upper panel) and pooled data (lower panel) of PP2AC in myocytes infected with control Adv (scrambled) or miR-1, or miR-133 or both miR-1 and miR-133; *<0.05 vs control, n = 6. H. Levels of miR-1 and miR-133 in myocytes assessed by qRT-PCR. *<0.05 vs control, n = 3. I. Infection of non-muscle cells (HEK) with miR-1 does not result in increase in expression levels of miR-133 and vise versa, *p<0.05 vs control, n = 3.

To elucidate the specific roles of miR-1 and miR-133 in the regulation of PP2A we infected rat ventricular myocytes with the corresponding adenoviral constructs. After 48 hours in culture myocytes infected with Ad-miR-133 exhibited ∼1.5-fold increase in Ca^2+^ transient amplitude in comparison with controls (Fig. 7A,B). Overexpression of miR-133 had no effect on SR Ca^2+^ content (Fig. 7A,C). Accordingly, the fraction of Ca^2+^ released from SR upon electrical stimulation, i.e. the ratio of Ca^2+^ transient amplitude to the amplitude of caffeine-induced transient was 50% higher in miR-133 infected myocytes than in control (Fig. 7D). These results suggest that enhanced excitation-contraction coupling in miR-133-overexpressing myocytes stems from increased responsiveness of RyR2s to triggering Ca^2+^, similar to what was previously described for miR-1 overexpressing myocytes [16]. Overexpression of miR-1 or miR-133 led to a significant increase in the frequency of pro-arrhythmic spontaneous Ca^2+^ waves in paced myocytes exposed to Iso. (Fig. 7E,F). Notably, coinfection of myocytes with both adenoviruses did not further augment spontaneous Ca^2+^ release. qRT-PCR studies revealed that myocytes infected with the miR-1 Adv increased not only miR-1 levels but the levels of miR-133 as well (Fig. 7H upper panel). A similar reciprocity was detected in miR-133 infected myocytes (Fig. 7H lower panel). Accordingly in three groups of myocytes; infected with miR-1 only, miR-133 only and miR-1 together with miR-133, Western blot experiments showed a similar 30% decrease in PP2AC with respect to control. Interestingly, in control experiments in non-muscle cells (HEK), only the miRNA carried by its respective viral construct was detected but not its counterpart (Fig. 7I). Taken together, these data suggest that in muscle cells, both miR-1 and miR-133 expression levels are coordinated.

### CaMKII inhibition attenuates diastolic Ca^2+^ waves underlying arrhythmogenic afterdepolarizations in HF myocytes

**Figure 8:**
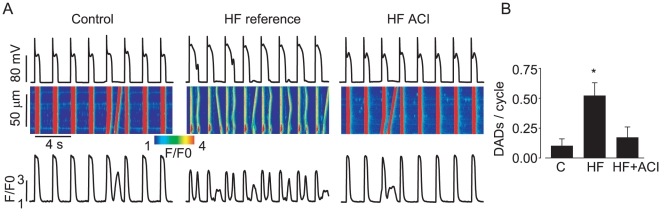
Inhibition of CaMKII activity attenuates pro-arrhythmic spontaneous Ca^2+^ waves in HF myocytes. A. Representative confocal Ca^2+^ images, with corresponding time dependent profiles (bottom) and membrane potential recordings (top) in myocytes isolated from normal and failing hearts in the presence of 100 nM Iso. B. Pooled data for a number of spontaneous Ca^2+^ waves per cycle. *p<0.05, n = 5–10.

To investigate the possible involvement of CaMKII in arrhythmogenesis in myocytes isolated from failing hearts we examined the effects of the specific CaMKII inhibitory peptide, ACI [31] on the generation of pro-arrhythmic spontaneous Ca^2+^ waves. Myocytes were paced in the current clamp mode (at 0.5 Hz) in the presence of Iso (100 nM). In stark contrast to control myocytes, HF myocytes exhibited spontaneous Ca^2+^ waves that caused delayed and early afterdepolarizations (Fig. 8A and 8B). Notably, introduction of the CaMKII inhibitory peptide (ACI, 200 µM) into the pipette solution restored normal periodic Ca^2+^ cycling in HF myocytes. To further assess the requisite role of altered CaMKII phosphorylation balance in pro-arrhythmic alterations of Ca^2+^ handling we examined the effects of the CaMKII inhibitor KN93 (1 µM, 15 min) on spontaneous Ca^2+^ waves induced on inhibition of PP2A in paced control myocytes from normal hearts in the presence of Iso. The inhibition of CaMKII in these experiments reversed the increase in arrhythmogenic propensity caused in myocytes by a combination of Iso and PP2A inhibition. (the number of spontaneous waves per cycle was 0.43±0.12 vs. 0.09±0.09* for Calyculin A (n = 20) and Calyculin + KN93 (n = 12), respectively, *statistically significant at p<0.05, Student's t-test). Thus, these results strongly support the central role of CaMKII-dependent phosphorylation in HF-induced Ca^2+^-dependent arrhythmogenesis.

## Discussion

In the present study, we have investigated the possible link between enhanced predisposition of ventricular myocytes toward pro-arrhythmic spontaneous Ca^2+^ release and altered expression levels of two major muscle-specific miRNAs, miR-1 and miR-133 in a canine model of chronic HF. Our main finding is that the increased expression of miR-1 and miR-133 observed in HF was associated with reduced levels of PP2A regulatory (B56α and B56δ) and catalytic subunits. The dissociation of PP2A activity from the RyR2 complex in HF resulted in CaMKII-dependent hyperphosphorylation of the RyR2 and profound pro-arrhythmic disturbances in Ca^2+^ cycling and membrane potential. Our results suggest that, in chronic HF, increased miR-1 and miR-133 expression levels lead to abnormal myocyte Ca^2+^ handling through disruption of site-specific PP2A phosphatase activity.

Our conclusions regarding the role of miR-1 and miR-133 in HF are supported by the following specific experimental findings: (1) pharmacological inhibition of PP2A activity in control myocytes was sufficient to promote proarrhythmic spontaneous Ca^2+^ release during β-adrenergic stimulation; (2) enhanced levels of miR-1 and miR-133 in HF myocytes were associated with (a) decreased expression of B56α and B56α known to be involved in subcellular targeting of PP2A and (b) reduced total expression levels of catalytic subunits of PP2A, reduced total PP2A activity and reduced levels of PP2A catalytic subunits associated with the RyR2; (3) in HF, the miRNA-associated reduction in PP2A levels led to significant increases in RyR2 phosphorylation at the PP2A-dependent sites Ser-2814 and Ser-2030; and (4) the functional consequences of pharmacological inhibition of phosphatases in control myocytes and of the miRNA-related decreases in total and localized PP2A activity in HF myocytes were reversed by CaMKII inhibitors.

HF is associated with pathological remodeling of ion channels and intracellular signaling pathways, including altered phosphorylation of RyR2s [8], [9]. Increased phosphorylation of RyR2s in HF has been suggested to arise from decreased activity of RyR2 channel-associated phosphatases [10], [11]. Consistent with these previous studies we found that the RyR2 is hyperphosphorylated at the CaMKII-dependent site, Ser-2814 (Fig. 6), and the expression of the catalytic and scaffolding regulatory subunits B56α and B56δ of PP2A are reduced in a canine tachypacing model of HF (Fig. 3). Additionally, in HF myocytes we found that RyR2 phosphorylation is increased at the site Ser-2030 (Fig. 6) which is tightly controlled by phosphatases [14] and this increase could be effectively reversed by pharmacological inhibition of CaMKII. In contrast, phosphorylation at the PKA site Ser-2808, which is known to be under the specific control of PP1, remained unchanged and coincided with unchanged levels of PP1 catalytic subunit (data not shown). Despite similar catalytic mechanisms, substrate recognition mechanisms for PP1 and PP2A are distinct. Emerging evidence suggests that recognition of specific Ser/Thr sites by PP2A involves additive effects of multiple distinct interactions [32], [33].

Recent studies have documented the importance of miRNAs in a number of pathological processes in the cardiovascular system, including cardiac arrhythmias [34], [35], [36], cardiac hypertrophy, HF, cardiac fibrosis and ischemia [20], [23], [24], [25], [26], [37], [38]. MiRNAs are a family of small, ∼21-nucleotides long, nonprotein-coding RNAs that have emerged as key post-transcriptional regulators of gene expression [17], [18]. Of the ∼1000 mammalian miRNAs discovered thus far, several appear to be muscle-specific and expressed in the heart, including miR-1 and -133 [28]. Genetic ablation of these two most abundant miRNAs in the heart has been reported to result in early death in transgenic animals, underscoring the vital importance of miR-1 and -133 for cardiac development and function [28]. Expression profiles of miR-1 and -133 have been shown to be dramatically altered in cardiac disease [20], [21], [22], [23], [24], [25], [26], [27] and are thought to play an important role in disease-related remodeling and arrhythmia [34], [35]. Here we demonstrate, for the first time, that miR-1 and miR-133 expression is coordinated; such that an increase in the level of one miRNA leads to increased expression of the other (Fig. 7). This result suggests that functionally miR-1 and miR-133 complement each other in such a way that the combination of mRNA targets inhibited by these miRNAs would result in pro-arrhythmic alterations in Ca^2+^ handling.

We recently reported that adenoviral-mediated overexpression of miR-1 increases CaMKII phosphorylation of the RyR2 by targeting the PP2A regulatory subunit B56α ~ Importantly, the decreased expression of B56α resulted in increased RyR2 activity and enhanced EC-coupling and promoted arrhythmogenic spontaneous Ca^2+^-release [16]. We hypothesized that this mechanism may be involved in RyR2 hyperphosphorylation and arrhythmogenesis in HF [16], [39]. Here we show, for the first time, that miR-1 and -133 are increased in a clinically relevant large animal model of HF [40]. Moreover, miR-1 and -133 overexpression was associated with reduced protein levels of the catalytic and regulatory subunits of PP2A, increased RyR2 phosphorylation and increased arrhythmic potential in HF myocytes. These alterations in Ca^2+^ cycling were mimicked by broad pharmacological inhibition of phosphatases (Fig. 1) and specific inhibition of PP2A (Fig. 5) in normal myocytes, whereas specific inhibition of CaMKII normalized Ca^2+^ cycling in cells isolated from failing hearts (Fig. 8).

The molecular mechanisms by which the expression of specific miRNAs are regulated is poorly understood, however, it is clear that the expression levels of miR-1 and miR-133 in the heart can differ depending on the stages and etiology of HF [41]. Future studies will carefully examine the dynamics of changes of miRNAs during disease progression as well as the possible effects of various therapeutic interventions on miRNAs expression profile. For example, a recent report showed that β-adrenergic antagonist (propranolol) can down-regulate miR-1 [42]. It is tempting to speculate that the antiarrhythmic effects of chronic administration of beta-blockers at least in part are stemming from their ability to regulate miRNAs and thus alter phosphatase activity in cardiac myocytes. The results presented here are also consistent with the previously published data suggesting that a shift in phosphorylation balance toward dephosphorylation in genetically modified mice [43] may have beneficial antiarrhythmic effects. In summary, these results demonstrate, for the first time, that increased RyR2 phosphorylation and arrythmogenesis in HF may result from miRNA-mediated decreases in PP2A activity. Our findings provide a rationale for establishing muscle-specific miR-1 and -133 as targets for antiarrhythmic therapy.

## Materials and Methods

### Heart Failure Model

All procedures were approved by The Ohio State University and Rhode Island Hospital Institutional Animal Care and Use Committees and conformed to the Guide for the Care and Use of Laboratory Animals published by the US National Institutes of Health (NIH Publication No. 85-23, revised 1996). A total of eight hound type dogs of either sex (2–3 years of age) had a right ventricular (RV) pacemaker lead implanted in the RV apex as previously described [44]. Following recovery from the pacemaker implant, the RV was paced at 180 BPM for 2 weeks; 200 BPM for the next six weeks, followed by 180 BPM for the duration of the protocol (modified Prevail 8086 pacemakers, Medtronic, Inc., Minneapolis, MN). Serial echocardiograms were performed at baseline and during brief periods of sinus rhythm during the pacing protocols, during butorphanol sedation (0.5 mg/kg, IM) [44]. A group of eight hound type dogs of either sex (2–3 years of age) served as controls.

### Myocyte cell isolation, cell culturing and transfection with adenoviruses

Dogs were euthanized with intravenous sodium pentobarbital or sodium thiopental, followed by rapid removal of the heart as previously described [44]. Hearts were rapidly excised via thoracotomy and perfused with cold cardioplegic solution (containing 5% glucose, 0.1% mannitol, 22.4 mM NaHCO3 and 30 mM KCl) injected into the coronary ostia. The left circumflex artery was cannulated for left ventricular mid-myocardial myocyte isolation. Following the washout of blood from the heart, collagenase (Worthington type II, 0.65 mg/ml) and protease-free bovine serum albumin (BSA) (0.65 mg/ml) were added to the perfusate (100 ml). After 30–45 min of collagenase perfusion, the digested mid-myocardial section of the lateral wall of the left ventricle was separated from the epicardial and endocardial sections; digested tissue was shaken in a water bath at 37°C for an additional 5–10 min. The myocytes were divided for molecular, biochemical, and electrophysiological assays and stored at room temperature until use. Isolation of rat ventricular myocytes was performed as previously described [45]. Isolated myocytes were plated on laminin-coated glass coverslips in serum-free medium 199 supplemented with (in mM): 25 NaHCO_3_, 5 creatine, 5 taurine, 10 U/ml penicillin, 10 µg/ml streptomycin, and 10 µg/ml gentamycin (pH 7.3). Cells were cultured at 37°C in a humidified atmosphere with 95% air/5% CO_2_. After 2 hours, unattached cells were removed and myocytes were infected with adenoviruses at multiplicity of infection (MOI) 100 and cultured for 36–48 hours before analysis. At such conditions culture-dependent changes in myocyte structure and function are minimal [46]. Recombinant adenoviruses were constructed, propagated, and tittered as previously described [24]. Briefly, pBHGloxΔE1,3Cre (Microbix), including the ΔE1 adenoviral genome, was co-transfected with the pDC shuttle vector containing the stem–loop sequence of the mouse *miR-1-2* and *miR-133a* gene, into 293 cells using Lipofectamine (Invitrogen, Carlsbad, CA). Through homologous recombination, the test genes integrate into the E1-deleted adenoviral genome. The viruses were propagated in 293 cells and purified using CsCl_2_ banding, followed by dialysis against 20 mM Tris buffered saline with 2% glycerol. Tittering was performed on 293 cells overlaid with DMEM plus 5% equine serum and 0.5% agarose.

### Ca^2+^ imaging and electrophysiology

Action potentials (APs) were recorded using the whole-cell patch clamp technique. The patch-clamp system was based on Axopatch 200B amplifier and DIGIDATA 1322A interface (Axon Instruments, CA). The external solution consisted of (mM): 140 NaCl, 5.4 KCl, 2.0 CaCl_2_, 0.5 MgCl_2_, 10 HEPES, and 5.6 glucose (pH 7.4). Patch pipettes were filled with the following solution (mmol/L): 90 K-aspartate, 50 KCl, 5 MgATP, 5 NaCl, 1 MgCl_2_, 0.1 Tris GTP, 10 HEPES, and 0.06 Fluo-3 (Molecular Probes, OR) (pH 7.2). The APs were evoked by application of 4-ms-long voltage pulses with amplitude 50% above the threshold level. Intracellular Ca^2+^ imaging was performed using an Olympus Fluoview 1000 and Leica SP5 confocal systems. Fluo-3 was excited by the 488 nm line of an argon-ion laser, and the fluorescence was acquired at wavelengths >510 nm. Rhod-2 was exited by 543 nm laser and the fluorescence was acquired at 590–690 nm wavelengths.

### Western Blot analysis, coimmunoprecipitation and CaMKII activity

Western blot analyses were performed as previously described [16] using RIPA buffer supplemented with phosphatase, calpain and protease inhibitors (Sigma, St Louis MO) and 1 µM Calyculin A (Calbiochem, Darmstadt, Germany) which was directly added to the cells after treatment to stop phosphorylation/dephosphorylation and the samples were instantly frozen in liquid N_2_. Cell lysate proteins were subjected to 4% to 20% SDS-PAGE and blotted onto nitrocellulose membranes (Bio-Rad Labs, Richmond CA). Primary antibodies used: anti-phospho-RyR2-S2808 and anti-phospho-RyR2-S2030 were raised against CRTRRIS(PO4)QTSQ-CONH2 and (CG)TIRGRLLS(PO4)LVEKVTYLKK-CONH2, respectively (Phosphosolutions, Aurora, CO). Anti-phospho-RyR2-S2814 was a gift from Dr. X. H. Wehrens (Baylor Coll. of Med., TX). For RyR2 phosphorylation, cells were studied before being lysed and were periodically field stimulated at 1 Hz for 1 min in Tyrode solution. Anti-RyR2, was from ThermoFisherScientific; anti-PP2A B56α, from Millipore (Billerica, MA); anti-PP2A B56δ from Abcam (Cambridge MA); anti-PP2AC from Calbiochem (Darmstadt, Germany); anti-GAPDH used as a loading control was from Abcam (Cambridge, MA). For coimmunoprecipitation the samples were incubated with protein A Sepharose beads at 4°C for 1 h, after which the beads were washed three times with buffer. Proteins (40–60 µg) were separated on SDS PAGE and blotted onto nitrocellulose membranes. Membranes were incubated for 1–2 hour at room temperature with primary anti-RyR2, anti-B56α or anti-PP2AC antibodies. Protein bands were visualized using the Super Signal West Pico kit (Pierce, IL).

Local CaMKII activity was measured in immunoprecipitated RyR2s samples using Cyclex non-radioisotopic kit according to manufacturer's instructions (CycLex Co., Columbia, MO). To perform the relative semi-quantitative immunoassay, the samples (10–20 µg) were diluted in Kinase Buffer, pipetted into microplate wells precoated with CaMKII substrate Syntide 2 and allowed to phosphorylate the bound substrate in the presence of Mg^2+^ and ATP. The amount of phosphorylated substrate was measured by binding with peroxidase conjugate of antibody that specifically detects phosphorylated substrate. The color was quantified by measuring absorbance at dual wavelength of 450/540 nm using microplate reader (Synergy MX, BioTeck Instruments Inc., Winooski, VT).

### Protein Phosphatase 2A activity assay

Tissue samples from the left-ventricle free wall were homogenized in buffer: 50 mM Tris-HCl, pH 7.5, 150 mM NaCl, 1 mM EDTA, 10% glycerol containing 1 mM DTT, 1 mM PMSF with complete protease inhibitors (Roche Diagnostics, Indianapolis, IN) using Tissue Tearor (Biospec Products, Inc). The homogenate was centrifuged at 15,000×g for 15 min. The clear supernatant was depleted of free phosphate by using Sephadex G-25 resin spin columns (Promega, Madison, WI). Protein Phosphatse 2A activity was determined using the serine/threonine phosphatase assay system microplate assay kit (Promega, Madison, WI), which determines the amount of free phosphate generated in a reaction by measuring the absorbance of a molybedate: malachite green: phosphate complex. Briefly, appropriate phosphate standards diluted in phosphate-free water, 5× PPase reaction mix and 1 mM phosphopeptide were added to a flat-bottom 96-well plate (total volume 50 µl). Reaction was initiated by adding protein sample (15–30 µg) following incubation at room temperature for 15–20 min. The reaction was stopped by adding equal volume (50 µl) of Molybedate dye, followed by incubation for 15–30 min at room temperature. Optical density of the molybedate: malachite green: phosphate complex was read using a plate reader with a 630 nm filter. Specific activity is expressed as picomoles of phosphate released/min/µg protein.

### Luciferase reporter constructs

A 1312-bp fragment encompassing the entire PP2A catalytic subunit α (PP2ACα) 3′-untranslated region (3′-UTR) was PCR-amplified utilizing the following sense (5′- TGA AAT TTT AAA CTT GTA CAG TAT TG -3′) and antisense (5′- GGT GAA TGT ACA TAA GAC TAA ATC -3′) primers. A 599-bp fragment encompassing the majority of the PP2A catalytic subunit β (PP2ACβ) 3′-UTR was PCR-amplified utilizing the following sense (5′- ATT TCT CCT GGG AAA CCT GCC TTT G -3′) and antisense (5′- GTT TAT TTG CTG AGT ACA CCA AAT AGG -3′) primers. Both were amplified using standard procedures and a proofreading polymerase (Platinum *Pfu*, Invitrogen). Human male genomic DNA (Promega) was used as template. Following the manufacturer's protocol, the PCR product was treated for 10 minutes with *Taq* polymerase. The PCR product was subsequently subcloned into the pCR™2.1 vector following the manufacturer's protocol (Invitrogen). Plasmid DNA was subsequently isolated from recombinant colonies and sequenced to ensure authenticity. The PP2ACα and PP2ACβ 3′-UTR inserts were removed from the pCR™2.1 plasmid by *Eco*RI digestion. The fragments were subsequently gel purified, filled in and blunt-end ligated into a filled-in *Xho*I site that is located downstream of the *Renilla* luciferase (r-luc) reporter gene (psiCHECK-2™, Promega). The authenticity and orientation of the inserts relative to the *Renilla* luciferase gene were confirmed by dideoxy sequencing. The resulting recombinant plasmids were designated, psiCHECK/PP2ACα and psiCHECK/PP2ACβ.

The mutant reporter construct psiCHECK/PP2ACα-mut was generated by utilizing the psiCHECK/PP2ACα plasmid as template and mutating the putative miR-133 recognition site (located at 122–128 bp) harbored in the PP2ACα 3′-UTR using the QuikChange site-directed mutagenesis kit (Stratagene). Briefly, a forward miR-133 mutagenic primer (5′- AAC TTG TTT TCA CAT **CCT GGT TT**A GAT GTG CCA TAT AAA AAT -3′) and a complementary reverse miR-133 mutagenic primer (5′- ATT TTT ATA TGG CAC ATC T**AA ACC AGG** ATG TGA AAA CAA GTT-3′), were synthesized and utilized in a PCR experiment as described by the manufacturer. The mutant reporter construct psiCHECK/PP2ACβ-mut was generated by utilizing the psiCHECK/PP2ACβ plasmid as template and mutating the putative miR-133 recognition site (located at 139–145 bp) harbored in the PP2ACβ 3′-UTR using the QuikChange site-directed mutagenesis kit (Stratagene). Briefly, a forward miR-133 mutagenic primer (5′- CAT TAA ACC ACA TCA T**CC TGG TTT** TGT GCC ATA CTA ATG ATG -3′) and a complementary reverse miR-133 mutagenic primer (5′- CAT CAT TAG TAT GGC ACA **AAA CCA GG**A TGA TGT GGT TTA ATG -3′), were synthesized and utilized in a PCR experiment as described by the manufacturer. Mutated sequence is shown in bold print. The amplification reactions were treated with *Dpn*I restriction enzyme to eliminate the parental template and the remaining DNA was used for transformation. The mutation of the miR-133a-1 seed binding sites was confirmed by dideoxy chain termination sequencing. Finally, transformed bacterial cultures were grown and each reporter construct was purified using PureLink™ Hipure Plasmid Maxiprep Kit (Invitrogen).

### Transfection and Luciferase assay

CHO cells were purchased from the American Type Culture Collection (ATCC, Manassas, VA) and maintained in Dulbecco's modified Eagle's medium (Invitrogen, Carlsbad, CA) supplemented with 10% fetal bovine serum (HyClone Laboratories, Logan, UT), 1× antibiotic-antimycotic, and 0.0175 mg/ml L-proline(Sigma). miR-133a-1 and negative control mimics (partially double-stranded RNAs that mimic the Dicer cleavage product and are subsequently processed into their respective mature miRNAs) were obtained from Dharmacon (Lafayette, CO). Transfection of CHO cells with small RNAs was optimized utilizing Lipofectamine 2000 (Invitrogen) and a fluorescein-labeled double-stranded RNA oligomer designated BLOCKiT™ (Invitrogen). Once conditions were optimized, CHO cells (approaching 100% transfection efficiency) were transfected with the luciferase reporter constructs described above and the appropriate miRNA precursor as indicated. After 24 hrs, CHO cells were washed and lysed with Passive Lysis Buffer (Promega), and firefly and *Renilla* luciferase activities were determined using the Dual-Luciferase Reporter Assay System (Promega) and a luminometer. *Renilla* luciferase expression in the psiCHECK vector is generated via an SV40 promoter. Additionally, the psiCHECK-2 vector possesses a secondary firefly reporter expression cassette which is under the control of the HSV-TK promoter. This firefly reporter cassette has been specifically designed to be an intraplasmid transfection normalization reporter; thus when using the psiCHECK-2 vector, the *Renilla* luciferase signal is normalized to the firefly luciferase signal.

### Real-time PCR

Total RNA was isolated from canine control and HF myocytes with Trizol reagent (Invitrogen). The RNA was subsequently treated with RNase-free DNase I, and mature miR-1 and -133 were quantified by utilizing TaqMan microRNA assay kits specific for each miRNA (Applied Biosystems, Foster City, CA) as previously described [16]. Briefly, 100 ng of total RNA was heated for 5 min at 80°C with 2.5 µM of the miR-1 or -133 and RNU48 antisense primers, followed by 5 min at 60°C then cooling to room temperature. The resulting solution was added to a cocktail and reverse transcription was performed in a 20 µl reaction according to the manufacturer's recommendations (Applied Biosystems). Quantitative real-time PCR (20 µl total reaction) was performed by using 5 µl of a 1∶5 dilution of cDNA. Results were analyzed using the 2^−ΔΔCt^ relative quantification method [47]. For each target miRNA, ΔC_t_ was calculated by subtracting their average C_t_ value from the average C_t_ value of the small noncoding RNA, RNU48.

### Data analysis

Echocardiographic data are presented as mean ± SD. Differences in echocardiographic parameters were evaluated by unpaired t-tests. Statistical significance of Ca^2+^ measurements and biochemical analyses were determined by one-way ANOVA and Student's t-test where appropriate and presented as mean ± SE.
